# 3D cinematic reconstructions of cardiovascular CT presented in augmented reality: subjective assessment of clinical feasibility and potential use cases

**DOI:** 10.1186/s41747-025-00566-1

**Published:** 2025-02-22

**Authors:** Benjamin Böttcher, Marly van Assen, Roberto Fari, Philipp L. von Knebel Doeberitz, Gabrielle Gershon, Felix G. Meinel, Carlo N. De Cecco

**Affiliations:** 1 Translational Laboratory for Cardiothoracic Imaging and Artificial Intelligence, Department of Radiology and Imaging Sciences, Emory University, Atlanta, USA; 2 Institute of Diagnostic and Interventional Radiology, Pediatric Radiology and Neuroradiology, University Medical Centre Rostock, Rostock, Germany; 3 Clinical and Experimental Medicine PhD Program, University of Modena and Reggio Emilia, Modena, Italy; 4 Institute of Clinical Radiology and Nuclear Medicine, University Medical Center Mannheim, Medical Faculty Mannheim of Heidelberg University, Mannheim, Germany; 5 Division of Cardiothoracic Imaging, Department of Radiology and Imaging Sciences, Emory University, Atlanta, USA

**Keywords:** Augmented reality, Cardiovascular system, Education (medical), Imaging (three-dimensional), Tomography (x-ray computed)

## Abstract

**Abstract:**

Augmented reality (AR) is a new technique enabling interaction with three-dimensional (3D) holograms of cinematic rendering (CR) reconstructions. Research in this field is in its very early steps, and data is scarce. We evaluated image quality, usability, and potential applications of AR in cardiovascular image datasets. Ten CR reconstructions of cardiovascular computed tomography (CT) datasets with complex anatomical abnormalities were presented to six radiologists and three cardiologists first on diagnostic screens and subsequently in AR. Subjective image quality and user experience were rated on 5-point Likert scales to assess usability and potential applications of AR. CR of CT datasets covering multiple images series of the same exam with differing kernels was performed in 143 ± 31 s (mean ± standard deviation); reconstruction of single CT image series took 84 ± 30 s. Mean subjective image quality was excellent, and observers showed high endorsement of the intuitive usability of the AR device and improvement of anatomical comprehensibility. AR devices were expected to have the greatest impact on patient and student education as well as multidisciplinary discussions, with less potential in clinical care. Clinical testing and preclinical implementation of AR seem feasible due to reasonable computation times and intuitive usability even for first-time users.

**Relevance statement:**

The presentation of 3D cinematic rendering in augmented reality provides excellent image quality, facilitating the comprehension of anatomical structures in CT datasets. Concurrently, reasonable computation times and the intuitive usability of augmented reality devices make preclinical implementation and clinical testing feasible.

**Key Points:**

- 3D cinematic reconstructions presented in augmented reality improve the anatomical comprehensibility of chest CT scans.
- Augmented reality devices are expected to be highly beneficial in educational settings and multidisciplinary discussions.
- Usability and computation times are feasible for initial preclinical use cases.

**Graphical Abstract:**

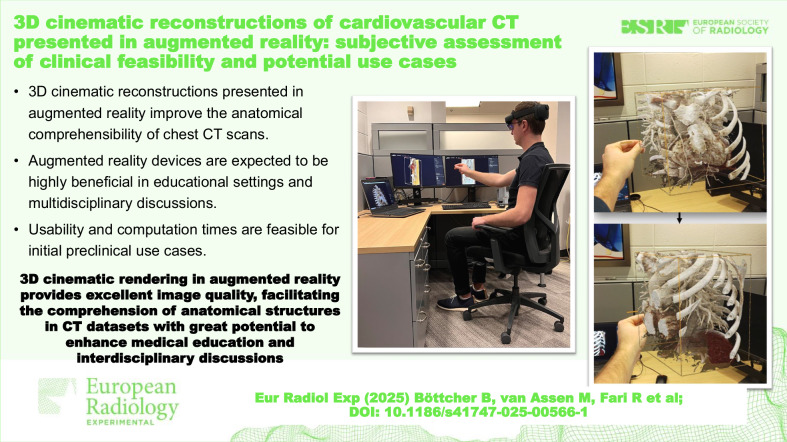

## Background

Comprehension of anatomical and pathophysiological conditions in patients is the cornerstone of medical education and resident training. Supported by the fact that nearly every subspecialized field in medicine requires diagnostic imaging for treatment planning, an ongoing effort is being taken to improve the comprehensibility of anatomical conditions. The cardiovascular system and its pathologies are highly related to structural changes and abnormalities peaking in complex congenital heart diseases.

Three-dimensional (3D) reconstruction of computed tomography (CT) images provides improved anatomical assessment, especially in complex compartments like the cardiovascular system. The current state-of-the-art reading applications use volume rendering (VR) techniques for 3D image reconstruction, where single-ray lightning models are utilized to calculate voxel-based 3D images. Cinematic rendering (CR) is a novel technique that uses similar calculation methods as VR but with advanced complex multi-ray lightning models [1–7]. CR calculations further integrate reflections and shadows from neighboring voxels providing photorealistic 3D imaging models with improved visualization compared to VR [8] or conventional CT source images [9].

Beyond CR image assessment on conventional diagnostic screens, augmented reality (AR) technology was recently introduced in medical imaging. AR enables interaction with computer generated 3D holograms within a real-world setting. Several AR devices have been developed, and literature is growing rapidly over the past years [10, 11]. Presentation of 3D CR images with AR offers unique ways of advanced interaction and comprehension of anatomic and pathologic conditions. Recent pilot studies indicate the beneficial value of AR device use in clinical applications [12, 13], but literature on this topic is still very scarce.

Therefore, this study aims to evaluate image quality, usability, and potential applications of an AR device presenting 3D holograms of cardiovascular image datasets.

## Methods

### Ethical approval and data retrieval

This retrospective single-center study was approved by the responsible Institutional Review Board, and the need for written informed consent was waived (see “Declarations” for details).

**Fig. 1:**
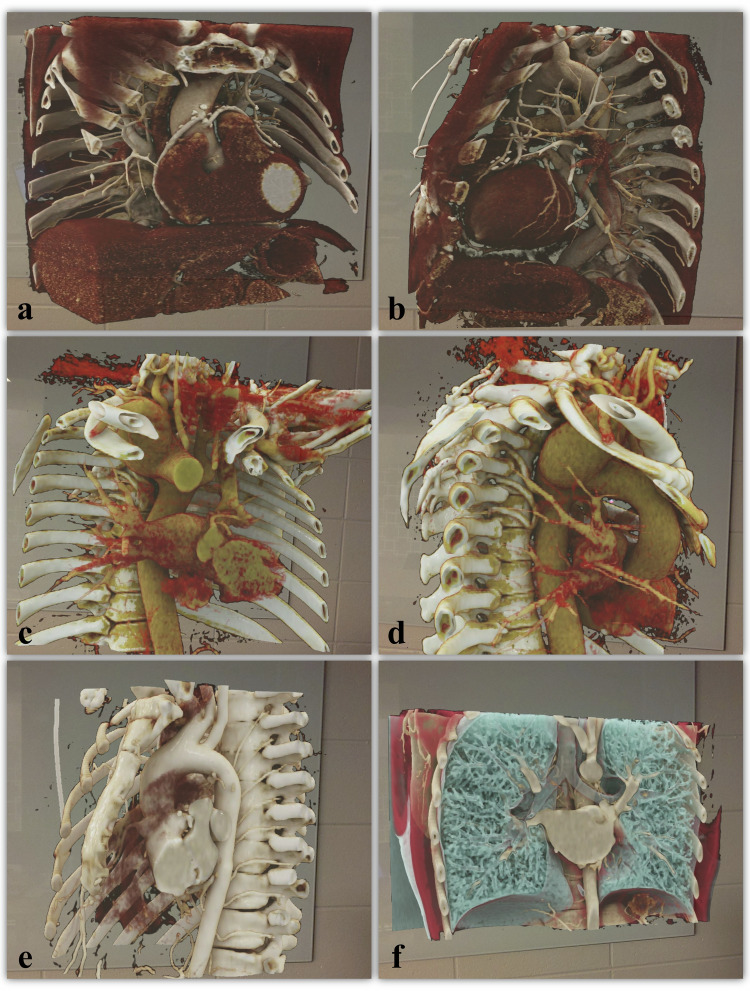
Visualization of 3D holograms from user perspective. Patient with multiple bypass grafts presented using coronary window preset in coronal perspective (**a**) and sagittal perspective (**b**). Patient with aortic arch anomaly presented using vascular window preset in coronal perspective (**c**) and sagittal perspective (**d**). Patient with separate left anterior descending artery and left circumflex artery origins presented in heart window preset in sagittal perspective (**e**) and in lung window preset in coronal perspective (**f**), highlighting the spatial relationship between vascular and airway structures

CT datasets were retrieved in a retrospective manner from the Picture Archiving and Communication System (PACS) focusing on cardiovascular CT cases with high complexity of anatomical abnormalities and changes. Patients of ≥ 18 years who underwent a chest CT scan between 2020 and 2022 were included. The diagnoses were verified by radiology reports and electronic health records. Datasets with impaired image quality (*e.g*., motion or respiratory artifacts) were excluded. Overall, ten contrast-enhanced CT datasets from five women and five men with mean age of 43.9 years (ranging from 23 to 65 years) were included. The images were acquired on two different CT systems: Somatom Force (nine exams) and Somatom Definition AS (one exam) (Siemens Healthineers, Erlangen, Germany). The following pathologies were included: aortic dissection (*n* = 2), coronary artery disease with multiple bypass grafts (*n* = 1), tetralogy of Fallot after surgery (*n* = 1), transposition of the great arteries (*n* = 2), Williams syndrome (*n* = 1), ascending aortic pseudoaneurysm (*n* = 1), and aortic arch anomalies (*n* = 2). Figure 1 visualizes example cases from the dataset.

### 3D cinematic reconstructions and augmented reality with HoloLens

**Fig. 2:**
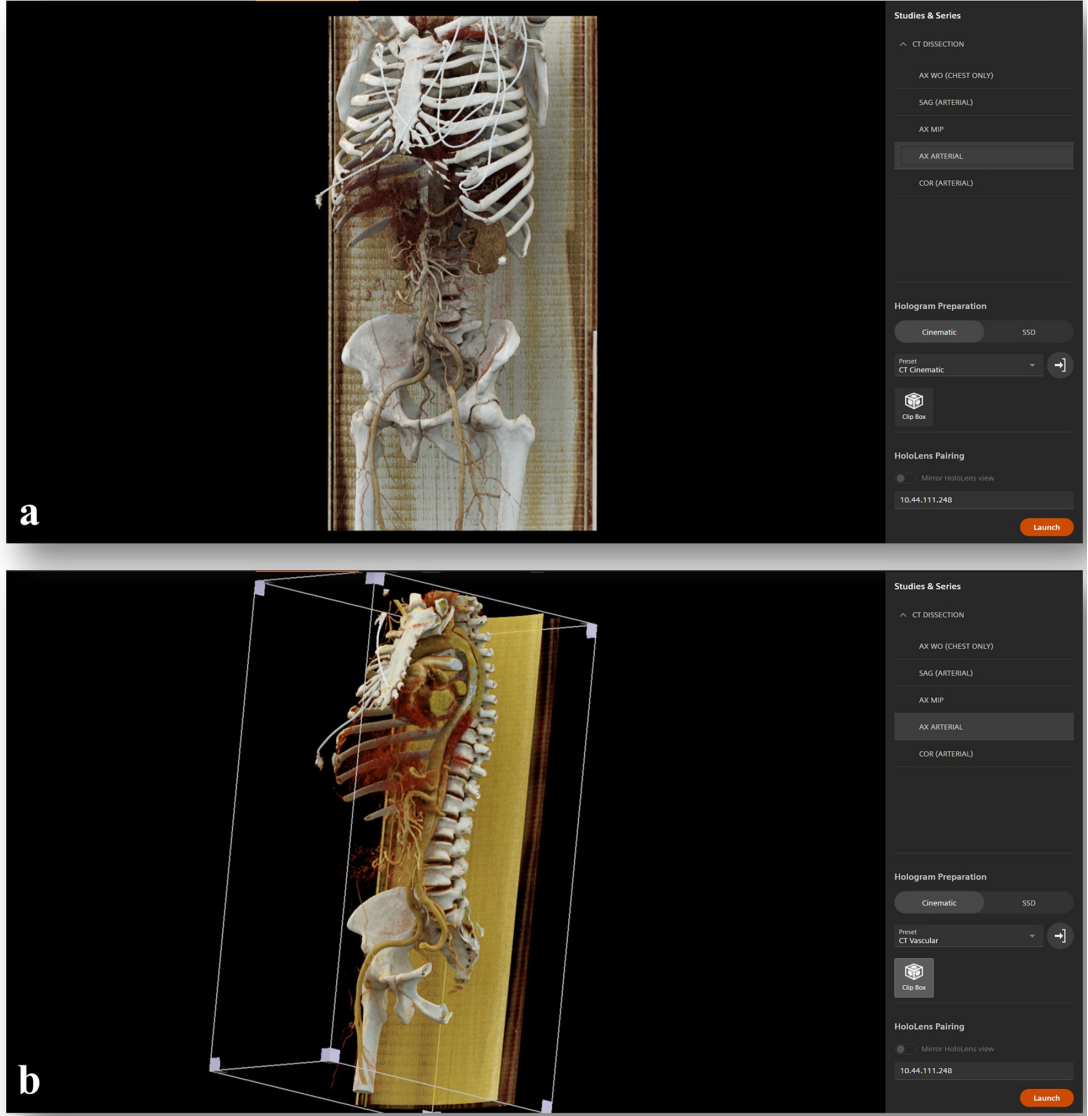
Cinematic reality software layout. **a** 3D cinematic reconstruction is shown in default setting. The menu on the right shows all available image series with 3D reconstructions as well as the presets which can be selected in a drop-down menu. **b** The same image series visualized with the vascular preset highlighting the vessels and cardiac structures. Additionally, the clip box is shown where the user can slice, rotate or zoom through the 3D model. 3D Three-dimensional

**Fig. 3:**
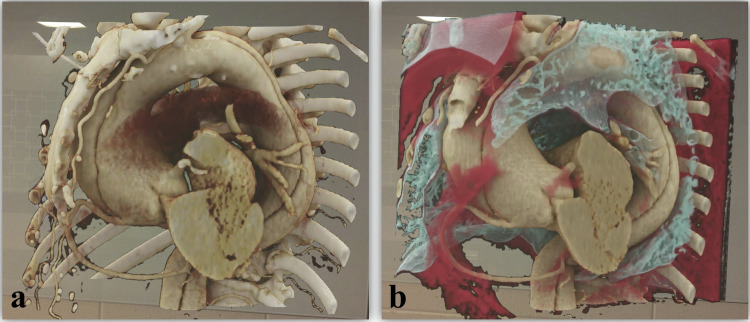
Visualization of a three-dimensional hologram from user perspective in different window presets. This case shows an aortic dissection type Stanford A with an ascending aortic aneurysm. **a** Aorta preset highlighting the aorta and main aortic branches in the chest. Soft and lung tissue are removed to improve depiction of aortic vascular structures. **b** Lung preset visualizing the lung tissue structure and adjacent soft tissue

Cinematic rendering was performed using a cinematic rendering software (Cinematic Reality, version 0.3.5.2, Siemens Healthineers, Erlangen, Germany) combined with a commercially available reading environment (syngo.via, version VB50, Siemens Healthineers, Erlangen, Germany). The rendering software was started by selecting single image stacks or whole CT datasets, including multiple image series with differing reconstruction kernels, and launch the rendering process in a drop-down menu. Figure 2 visualizes the software layout and two of many available presets. After the calculation was completed, the 3D model was visualized in a separate window. Soft tissue transparency and contrast could be modified, as preferred. Several predefined settings were provided to enhance certain features, such as vessels, bones, or soft tissue. Figure 3 provides an example of a 3D hologram in different window presets.

**Fig. 4:**
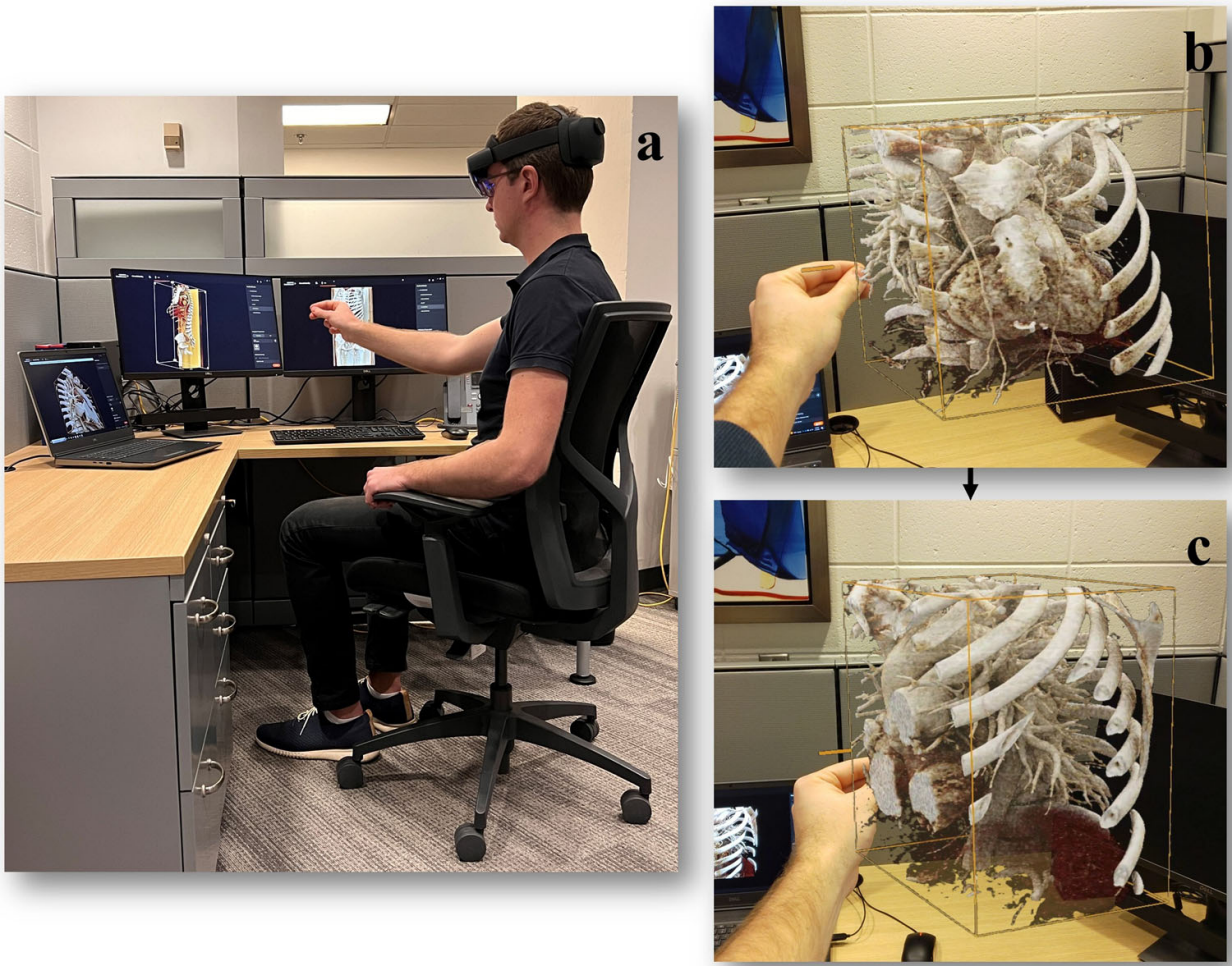
Visualization of HoloLens use case. **a** Example of a desk set up for HoloLens use. **b**, **c** Three-dimensional hologram from the user perspective. The clip box is used to interact with the hologram by hand gestures, in this example, the slicing function is shown

An augmented reality device (HoloLens 2, Microsoft Corp., Seattle, USA) was paired with the CR software using a Wi-Fi connection to launch 3D reconstructions in AR. Hand gestures were used to zoom, rotate or slice the hologram, whereas window settings needed to be selected on the paired computer. A Supplementary Video clip (S3) visualizes the hologram including the available interactions from the user’s perspective. Figure 4 shows the AR device in a use case from the user’s perspective.

### Study layout

The study was conducted in single test sessions. Test sessions were completed by nine observers including three cardiologists (one resident and two attendings) and six radiologists (one resident and five attendings) with clinical CT reading experience (not limited to cardiovascular CT examinations) ranging from 1 to 18 years. Cardiologists had a mean experience of 2.3 years of clinical CT reading (range 1–5 years) whereas radiologists had a mean of 10.2 years of clinical CT reading (range 4–18 years).

During sessions, CT axial thin sliced datasets with a soft tissue kernel were presented first using the conventional reading platform including standard CT multiplanar reconstructions and 3D cinematic rendering without augmented reality. Subsequently, observers used the AR device to assess the same 3D cinematic reconstructions in augmented reality. Readers were encouraged to interact with the hologram and use all functions of the AR device as preferred. Afterward, readers were asked to rate subjective image quality after each CT case on a 5-point-Likert scale from 1 (= “incomprehensible”) to 5 (= “excellent”). At the end of each session, user experience-based statements were rated on a 5-point Likert scale from 1 (= “definitely not”) to 5 (= “definitely yes”). Supplementary Table S1 summarizes all ratings during the test sessions. The case-based ratings (Q1–Q8) aimed to assess the subjective image quality of holograms, whereas experienced-based statements (Q9–Q16) were designed to evaluate the usability and potential applications of the AR device.

The time used for 3D cinematic reconstruction was recorded for whole CT dataset rendering and single series rendering.

### Statistical analysis

Statistical analysis was performed using SPSS statistics, version 29.0.0 (IBM Corporation, Armonk, NY, USA). Observer ratings were analyzed overall and in the following subgroups: experience-based (residents (*n* = 2) *versus* attending physicians (*n* = 7)) and profession-based (radiologists (*n* = 6) *versus* cardiologists (*n* = 3)).

## Results

### Processing time and subjective image quality of 3D holograms

Mean calculation time for cinematic rendering of whole 2D CT datasets covering between 2 and 5 axial thin sliced images series from the same exam with different reconstruction kernels (*e.g*., soft tissue kernel, lung kernel, bone kernel) was 143 ± 31 s (mean ± standard deviation). Computation of CR images from single image series with soft tissue kernel was performed in 84 ± 30 s.

The results of subjective image quality ratings of holograms are summarized in Supplementary Table S2. Excellent median image quality ratings were observed for overall impression, depiction of great vessels, bones, and mediastinal structures. Cardiac structures (including coronary vessels and ventricle structures) and visualization of underlying pathologies reached very good median ratings. Experienced-based subanalysis showed that residents tended to score higher median ratings for hologram image quality (median of 5 for Q1–Q8) compared to attending physicians (median of 5 in Q1/Q2/Q6, 4.5 in Q5, and 4 in Q3/Q4/Q7/Q9).

Profession-based subanalysis revealed that radiologists tended to score slightly higher image quality ratings compared to cardiologists.

### Usability and potential applications of AR device

Supplementary Table S2 provides an overview of user experience ratings and potential applications of AR devices. Observers gave excellent median ratings for intuitive use of the AR device; its benefit for increased comprehensibility over CR presented on conventional monitors alone. Median ratings of 4 (= “probably yes”) were given for AR device, adding important information for cardiac imaging.

The expected benefit of using 3D cinematic reconstructions with AR devices was rated highest in the field of student and patient education, as well as in multidisciplinary discussions like cardiac board meetings. The clinical care benefit was considered lower compared to other applications.

Experience-based subanalysis revealed no differences between residents and attendings whereas cardiologists tended to rate the clinical care benefit higher compared to radiologists.

## Discussion

This study evaluated the usability and potential applications of a novel AR device presenting 3D cinematic reconstructions of cardiovascular CT datasets by assessing subjective image quality, usability, and potential benefit in predefined use cases. Mean calculation time for 3D cinematic rendering showed reasonable results acceptable for routine use. Observers showed high consent for the intuitive usability of the AR device and excellent subjective image quality of holograms, improving anatomical comprehensibility. The highest benefit of AR device application was expected for educational purposes and multidisciplinary discussions such as board meetings, while the expected benefit in daily clinical care was rated limited.

Calculation time is an important criterion for the clinical usability of novel technologies. In our study, mean reconstruction times for 3D cinematic rendering of CT datasets required for AR device use did not extend over 4 min. Considering the intuitive use of the AR device even for first-time users, it could be stated that presenting 3D reconstructions in AR is technically feasible for clinical use in certain settings. Moreover, preselection of a single thin sliced image series, for example, with a soft tissue kernel, rather than run the cinematic reconstruction on all image series with different kernel settings at once, and expected future improvements of software and hardware components promising significant reduction of processing times suitable for routine clinical use.

Our study showed excellent subjective image quality ratings of holograms with high consent between observers. This is in line with a publication from Gehrsitz et al [12], who used holograms of 3D cinematic reconstructions for pediatric heart surgery planning and reported a significant reduction of pre- and intraoperative planning time. This underlines that 3D cinematic rendering presented in augmented reality is most valuable for improving the comprehension of spatial relationships of anatomical structures critical for surgical planning or diagnosis of complex congenital cardiovascular anomalies. Interestingly, in our study cardiac structures such as coronary arteries and inner heart structures were rated with lower image quality. This was also found by Gehrsitz et al [12]. These findings suggest that the depiction of submillimeter structures or composition of thoracic organs in holograms is limited. Consequently, holograms of 3D CR are not suitable for diagnostic use yet. Further, current standard diagnostic tools used for CT image interpretation, such as distance measurements or HU-value measurements, are not available for the AR device. The introduction of novel scanner technologies, such as photon-counting detectors, might enhance the quality of the 3D CR holograms to a level suitable for diagnostic use in the future.

Nevertheless, cardiologists participating in our study reported higher expected benefit of AR device use for clinical care and decision-making compared to radiologists. Interestingly, surgeons interviewed by Gehrsitz et al reported a similar benefit for planning of cardiovascular surgery in pediatric patients [12] impacting clinical care. Another preclinical study assessing presurgical planning using cinematic rendering without AR by Elshafei et al showed improved anatomical comprehension and reduced preparation times [14]. These findings indicate that especially non-radiologist professions benefit from AR image presentation. This is most likely due to the fact that radiologists are trained to interpret 2D HU-value-based CT images while other professions like cardiologists and surgeons are not. Additionally, these differences in comprehension of CT data are a major challenge in interdisciplinary board meetings. A recently published case series from Recht et al provides first-use cases in pediatric patients with thoracic vascular anomalies, highlighting the benefit for interdisciplinary treatment planning [15]. The application of AR devices can elevate these advancements to a new level by adding a real 3D perspective. While this benefit can be expected to be high among experienced clinicians, resident training and patient education for the informed consent process can benefit even more from AR device use.

Our study has limitations that must be mentioned. First, this study was conducted with only a small number of CT datasets in a single center. To evaluate clinical and diagnostic impact on the proposed applications in clinical care further large cohort studies are needed with special focus on diagnostic accuracy and time efficiency. Larger cohorts will also allow verifying whether the differences observed are statistically significant. Second, the test setting in our study was optimized for AR device use. More testing with different technical setups is needed to prove clinical usability.

In conclusion, this study showed that 3D cinematic rendering presented in augmented reality provided excellent subjective image quality adding important information for anatomical comprehension. Clinical testing and preclinical implementation of AR devices seem feasible due to reasonable computation times and intuitive usability even for first-time users. Observers expect a high benefit of AR device use in educational settings and multidisciplinary discussions such as board meetings.

## Supplementary information

**Additional file 1: Supplementary Table S1**. **Supplementary Table S2**.

Supplementary Videoclip

## Data Availability

The datasets used and/or analyzed during the current study are available from the corresponding author upon reasonable request.

## Abbreviations

3D: Three-dimensional
AR: Augmented reality
CR: Cinematic rendering
CT: Computed tomography
VR: Volume rendering
