# Supplementary material for: 3D cinematic reconstructions of cardiovascular CT presented in augmented reality: subjective assessment of clinical feasibility and potential use cases
Source: Eur Radiol Exp. 2025 Feb 22;9:27. doi: 10.1186/s41747-025-00566-1 (PMC11846813; doi:10.1186/s41747-025-00566-1)
Supplement: Supplementary file 1 — Additional file 1: Supplementary Table S1. Supplementary Table S2. [file 41747_2025_566_MOESM1_ESM.pdf]

3D cinematic reconstructions of cardiovascular CT presented in augmented reality: subjective assessment of clinical feasibility and potential use cases

ELECTRONIC SUPPLEMENTARY MATERIAL

Supplementary Table S1

Number	Question/Statement	Scale
**Image quality of 3D cinematic rendering presented in augmented reality**		
**Q1**	Overall spatial impression	1 – incomprehensible<br>2 – poor<br>3 – somewhat good<br>4 – very good<br>5 – excellent
**Q2**	Comprehensibility of aorta relationship	
**Q3**	Comprehensibility of pulmonary spatial relationship	
**Q4**	Comprehensibility of spatial relationship between cardiac structures	
**Q5**	Comprehensibility of spatial relationship between pulmonary structures	
**Q6**	Comprehensibility of spatial relationship between osseus structures	
**Q7**	Comprehensibility of spatial relationship between mediastinal structures	
**Q8**	Comprehensibility of underlying pathology	
**User interaction with augmented reality device**		
**Q9**	The HoloLens was easy and intuitive to use with the given instructions.	1 – definitely not<br>2 – probably not<br>3 – maybe<br>4 – probably yes<br>5 – definitely yes
**Q10**	The HoloLens may add important information that warrants the additional time spend for its use.	
**Q16**	The HoloLens provides better understanding in comparison with cinematic rendering.	
**Applications of 3D cinematic rendering presented in augmented reality**		
**Q11**	The HoloLens would be useful in clinical care and clinical decision making.	1 – definitely not<br>2 – probably not<br>3 – maybe<br>4 – probably yes<br>5 – definitely yes
**Q12**	The HoloLens would be useful for resident training or student education.	
**Q13**	The HoloLens would be useful for patient education and the informed consent process.	
**Q14**	The HoloLens could improve communication in multidisciplinary discussions.	
**Q15**	The HoloLens would be useful for tumor board or cardiac board meetings.	

Questions asked during each test session. The case-base questionnaire contained Q1 – Q8 whereas Q9 – Q16 were asked at the end of each session.

Supplementary Table S2

Question	Median rating	Lowest rating	Highest rating
**Image quality of 3D cinematic rendering presented in augmented reality**			
**Q1 overall impression**	5 (4 – 5)	2	5
**Q2 aorta**	5 (5 – 5)	4	5
**Q3 pulmonary relationship**	5 (3.75 – 5)	1	5
**Q4 cardiac structures**	4 (3 – 5)	1	5
**Q5 pulmonary structures**	5 (4 – 5)	2	5
**Q6 osseus structures**	5 (5 – 5)	3	5
**Q7 mediastinal structures**	5 (3 – 5)	2	5
**Q8 pathology**	4 (4 – 5)	1	5
**User interaction with augmented reality device**			
**Q9 intuitive use**	5 (4 – 5)	4	5
**Q10 add information**	4 (3 – 4)	3	5
**Q16 improves understanding**	5 (3.5 – 5)	3	5
**Applications of 3D cinematic rendering presented in augmented reality**			
**Q11 clinical care**	4 (3 – 4)	2	5
**Q12 student education**	5 (5 – 5)	4	5
**Q13 patient education**	5 (4 – 5)	4	5
**Q14 multidisciplinary discussions**	5 (4 – 5)	3	5
**Q15 tumor/cardiac board**	5 (3.5 – 5)	3	5

Median, lowest and highest ratings for all asked questions. Medians are shown with IQR.

Supplementary Videoclip

Visualization of a hologram from a 3D reconstruction in the cardiovascular preset. Hand gestures were used to zoom, rotate or slice the hologram, whereas window settings needed to be selected on the paired computer.
